# Induced swimming in European seabass (*Dicentrarchus labrax*): effects on the stress response, immune, and antioxidant status

**DOI:** 10.1007/s10695-025-01474-2

**Published:** 2025-03-03

**Authors:** Carlos Espírito-Santo, Francisco A. Guardiola, Rodrigo O. A. Ozório, Leonardo J. Magnoni

**Affiliations:** 1 Faculty of Sciences, University of Porto, Rua Do Campo Alegre, 4069-007 Porto, Portugal; 2 Interdisciplinary Centre of Marine and Environmental Research (CIIMAR), Terminal de Cruzeiros Do Porto de Leixões, University of Porto, Av. General Norton de Matos S/N, 4450-208 Matosinhos, Portugal; 3 Immunobiology for Aquaculture Group, Department of Cell Biology and Histology, Faculty of Biology, Campus Regional de Excelencia Internacional “Campus Mare Nostrum”, University of Murcia, 30100 Murcia, Spain; 4 The New Zealand Institute for Plant and Food Research Limited, 293 Akersten St. Port Nelson, Nelson, 7010 New Zealand

**Keywords:** Swimming, Stress response, Immune response, Oxidative stress, European seabass (*Dicentrarchus labrax*), Fish welfare

## Abstract

Suitable swimming conditions can improve the growth and welfare of farmed fish. This study investigated how swimming affects immune and oxidative responses in European seabass (*Dicentrarchus labrax*), an important farmed fish species in Southern Europe. Thirty-two specimens were assigned into four experimental groups with the following conditions for 6 h: steady low (L, 0.8 body lengths (BL)⋅s^−1^); steady high (H, 2.2 BL⋅s^−1^); oscillating (O, 0.8–2.2 BL⋅s^−1^) swimming speeds; and control non-induced to swim (C, < 0.1 BL⋅s^−1^). The H group exhibited higher white blood cell counts and plasma cortisol levels compared to the C and L groups. However, innate immune parameters in plasma and skin mucus showed no differences between groups. Gene expression revealed an up-regulation of inflammatory cytokines (*tnfα* and *il1β*) and *igf1* in the gills of fish from H and L groups, respectively, whilst no variations were observed in the head-kidney. In the skin, *tnfα* was up-regulated in the L group. Moreover, the H group showed increased superoxide dismutase and catalase activities and higher lipid peroxidation levels in red muscle. The L group had a higher ratio of reduced/oxidized glutathione (GSH: GSSG) in red muscle, suggesting enhanced antioxidant status. Under all swimming conditions, the GSH: GSSG ratio was increased in the white muscle. Conversely, hepatic markers of oxidative stress were similar among groups. Results suggest that steady swimming at 0.8 BL⋅s^−1^ enhanced the antioxidant status in red muscle which may be of relevance to improve the welfare of this cultured species.

**Supplementary Information:**

The online version contains supplementary material available at 10.1007/s10695-025-01474-2.

## Introduction

In farmed conditions, fish can be induced to swim by creating suitable water currents. Previous studies in fish have investigated how induced swimming activity can change growth and sexual maturation, modulating as well neural plasticity and stress response, with a potential impact on fish welfare (McKenzie et al. 2021). In mammals, exercise has been shown to modulate the immune system and antioxidant status (Campbell and Turner 2018; Powers et al. 2020). In fish, some studies have investigated the relationship between induced swimming activity and welfare parameters, such as immune and antioxidant status. For example, induced swimming activity has been shown to improve the immune status in Atlantic salmon (*Salmo Salar*) (Castro et al. 2011), *Percocypris pingi* (Li et al. 2023), rock carp (*Procypris rabaudi*) (Hou et al. 2022), and in ya-fish (*Schizothorax prenanti*) (Liu et al. 2018). However, the response is highly dependent on the fish species and swimming conditions, as no effects (Espírito-Santo et al. 2023) or even negative effects (Zhu et al. 2023) of induced swimming on the immune status have been described. Similarly, induced swimming can affect the antioxidant status of fish, particularly in the liver and skeletal muscle (Aniagu et al. 2006; Sánchez-Moya et al. 2022; Espírito-Santo et al. 2024a). However, to our knowledge, the understanding of the effects of induced swimming activity in such parameters in European seabass (*Dicentrarchus labrax*) is still very limited. The European seabass is a perciform species widely produced in Southern Europe, targeted to increase production while including fish welfare considerations (FAO 2024). This species is a body caudal fin swimmer (Marras et al. 2013), able to maintain its position in strong currents (Handelsman et al. 2010). This species undertakes seasonal migrations between inshore feeding and offshore spawning areas and relies on fast sprints to capture its prey and avoid predators (Kelley and Pickett 1987). Therefore, it can effectively perform both short anaerobic sprints and sustained aerobic swimming (Marras et al. 2013). The swimming physiology of the European seabass has been investigated in combination with hypoxia (Zhang et al. 2017), food deprivation (Dupont-Prinet et al. 2010), or temperature changes (Stavrakidis-Zachou et al. 2022).

Reduced water flow and high stocking densities can alter the natural swimming behavior of fish reared in recirculating aquaculture system (RAS) tanks, ponds, or sea cages (Muñoz et al. 2020). The effects of intensive aquaculture conditions on fish swimming behavior have been studied mainly in salmonid species, which generally experience higher water flow in the wild and adapt their swimming activity to the prevailing conditions (Palstra and Planas 2011). Therefore, the stimulation of natural swimming behavior should be considered when designing the enclosures, which may allow for enhancing the performance and welfare of farmed fish.

The current study aimed to investigate the effects of 6 h of induced swimming in different conditions on the welfare of European seabass by evaluating stress, innate immune, and antioxidant responses. This duration was used as it is an adequate method to verify the underlying mechanisms regulating the physiological effects of induced swimming activity on immune-related parameters and oxidative stress status, by analogy to what has been studied in mammals (Yang et al. 2005; Bessa et al. 2016; Nieman and Wentz 2019), due to the lack of studies in fish exploring this potential link. Furthermore, this experimental setting allows the minimization of the effects that confounding factors, such as feeding and social interactions, may have on the physiology and behavior of fish when applied in long-term trials.

## Materials and methods

### Experimental design

The Ethics Committee of the University of Murcia approved the experimental protocols following the European Union guidelines for animal handling (2010/63/EU). European seabass juveniles were obtained from a fish market (Piscicultura Marina Mediterránea, SL, Castellón, Spain), and kept in an 800 L tank connected to a RAS at the Marine Fish Facilities (University of Murcia) for 20 days before the start of the experimental trial. The water temperature was maintained at 20.0 ± 1.0 °C with a flow rate of 900 L⋅h^−1^ and 30‰ salinity. The photoperiod was 12 h light and 12 h dark, and the fish were fed a commercial pellet diet (Skretting) at a rate of 2% body weight per day. Ammonia and nitrite levels in the water were measured twice a week using commercial kits (MultiTest, Seachem) and never exceeded 0.025 and 0.3 mg⋅L^−1^, respectively.

### Swimming trials

Thirty-two European seabass (total length: 11.5 ± 0.1 cm; body weight: 15.5 ± 0.6 g) were assigned to the following four conditions: (i) steady low (L, 0.8 body lengths (BL)⋅s^−1^); (ii) steady high (H, 2.2 BL⋅s^−1^); (iii) oscillating (O, 0.8–2.2 BL⋅s^−1^) swimming speeds; and (iv) non-exercised control group (C, < 0.1 BL⋅s^−1^). The swimming protocols were applied following the study by Espírito-Santo et al. (2024b), adapted from Graziano et al. (2018). In brief, fish were individually housed in swimming flumes for 24 h for acclimatization with a slow current set to circulate the water inside the tank and within the swimming flume (approximately 1 cm⋅s^−1^), followed by 6 h in one of the four conditions described (*n* = 8). The flumes were connected to a propeller regulated by an electronic controller (EcoDrift 20.1, AquaMedic) at one end of the tube. The water was channeled through a flow rectifier (honeycomb) to generate a laminar flow. All flumes contained a grid at the end to prevent the fish from being washed away. The oscillating swimming condition generated by the propeller consisted of a controlled change in water flow of 5 cm⋅s^−1^ every 2 s between minimum and maximum speeds.

### Sampling

The fish were anesthetized with 3-aminobenzoic acid ethyl ester (MS-222; 0.1 g⋅L^−1^) buffered in NaHCO_3_ (0.2 g⋅L^−1^) immediately after the end of each trial. The skin mucus was then collected by carefully scraping the dorsolateral surface with a cell scrapper, centrifuged (2000 × *g*, 10 min, 4 °C), and stored at − 80 °C, according to Guardiola et al. (2014). The blood was withdrawn via the caudal vessels with a heparinized syringe and used to determine the total count of red blood cells (RBC) and white blood cells (WBC) using a hemocytometer (Marienfeld). The remaining blood was used to obtain plasma by centrifugation (10,000 × *g*, 5 min) and stored at − 20 °C. Subsequently, fish were euthanized with an overdose of MS-222 (100 mg⋅ml^−1^ water). The head-kidney (HK), gills (first and second arch), and skin were collected for gene expression analysis. Finally, skeletal red and white muscle from the caudal and dorso-anterior region, respectively, and liver were sampled for enzymatic assays and immediately frozen at − 80 °C.

### Stress markers in plasma

Plasma glucose and lactate concentrations were determined using commercial kits based on enzymatic colorimetric assays (GOD-POD and LO-POD, SPINREACT). Cortisol concentrations were determined using an enzyme immunoassay kit based on the competitive link between cortisol and related monoclonal antibodies (RE 52061, IBL International). The kit includes multiple reference samples with established cortisol concentrations, designed to construct a standard curve, and was previously validated for European seabass by Azeredo et al. (2017). All measurements were performed in triplicate and according to the manufacturer’s recommendations.

### Immune parameters in plasma and skin mucus

Natural hemolytic complement activity was measured in plasma according to the protocol of Sunyer and Tort (1995), using rabbit red blood cells (RaRBC, Probiologica Lda). The ACH_50_ units were defined as plasma concentration that induces 50% hemolysis of RaRBC. Lysozyme activity in plasma and skin mucus was measured by a turbidimetric method (Swain et al. 2007) based on the lysis of *Micrococcus lysodeikticus* (0.2 mg⋅mL^−1^, Sigma-Aldrich) by hen egg white lysozyme (HEWL, Sigma). Lysozyme activity was expressed as U⋅mL^−1^ equivalent of HEWL activity. Peroxidase activity in plasma and skin mucus was calculated using the 3,3′,5,5′-tetramethylbenzidine hydrochloride (Sigma) reduction assay by Quade and Roth (1997). Bactericidal activity in skin mucus was determined using two opportunistic marine pathogenic bacteria (*Vibrio anguillarum* and *Photobacterium damselae*), as described by Guardiola et al. (2019). Bactericidal activity was expressed as the percentage of non-viable bacteria, calculated as the difference between the absorbance of surviving bacteria compared to the absorbance of bacteria from the positive controls (100%). Due to the limited plasma sample volume, bactericidal activity was only measured in skin mucus.

### Gene expression in head-kidney, gills, and skin

Total RNA was extracted from HK, gills, and skin of European seabass using TRIzol™, according to the manufacturer’s instructions, purified and quantified using a Nanodrop® spectrophotometer. RNA was treated with DNase I (Promega) to eliminate genomic DNA contamination, and complementary DNA (cDNA) was synthesized from 1 μg of RNA using the reverse transcriptase enzyme SuperScript IV (Life Technologies) with an oligo-dT_18_ primer. The expression of the selected genes (Table 1) was analyzed by real-time qPCR using QuantStudio™ Real-Time PCR System Fast (Life Technologies). Gene expression was calculated using the 2^−ΔCt^ method (Livak and Schmittgen 2001). The specificity of the reactions was analyzed using samples without cDNA as negative controls and for each gene. Gene expression was normalized with the expression of elongation factor 1-alpha (*ef1α*) in each sample. The primers used for each gene analysis were designed using the Thermo Fisher OligoPerfect™ tool, and their specificity was assessed by melt curves in which the values of efficiency were between 90 and 110%.

**Table 1:** Primers used for qPCR

Gene name	Gene abbreviation	Accession number	Primer sequences (5′ → 3′)
Elongation factor 1-alpha	*ef1α*	AJ866727	F: CGTTGGCTTCAACATCAAGA R: GAAGTTGTCTGCTCCCTTGG
Tumor necrosis factor-alpha	*tnfα*	DQ200910.1	F: CGAGGGCAAGACTTTCTTTG R: GCACTGCCTGTTCAGCTACA
Interleukin 1 beta	*il1β*	AJ269472	F: CAGGACTCCGGTTTGAACAT R: GTCCATTCAAAAGGGGACAA
Interleukin 6	*il6*	AM490062	F: ACTTCCAAAACATGCCCTGA R: CCGCTGGTCAGTCTAAGGAG
Nuclear factor kappa B	*nfκb*	DLAgn_00239840	F: GCTGCGAGAAGAGAGGAAGA R: GGTGAACTTTAACCGGACGA
Insulin growth factor 1	*igf1*	GQ924783	F: AGATGTACTGTGCACCTGCC R: CTTTGTGCCCTGCGGTACTA
Lysozyme	*lyz*	FN667957	F: ATTTCCTGGCTGGAACACAG R: GAGCTCTGGCAACAACATCA
Carbonic anhydrase	*cahz*	AJ854106	F: GATGGAAAGCGCTATCCCATGGAGTTACA R: CCTGTAAACTTAGTGCAAGTGCATTCCTGCC

### Metabolic enzymes

Frozen red and white skeletal muscle were powdered using mortar and pestle cooled with liquid N_2_ and divided into aliquots for metabolic and oxidative stress enzyme assays, before homogenization. For metabolic enzymes, red and white skeletal muscle samples were homogenized as described by McClelland et al. (2005). The activity of citrate synthase (CS) was determined with oxaloacetate as substrate according to McClelland et al. (2005). The activity of lactate dehydrogenase (LDH) was measured by the rate of NADH consumption with pyruvate as substrate (McClelland et al. 2005). Changes in absorbance were measured at 25 °C in a microplate reader (BioTek Instruments, Inc.).

### Antioxidant enzymes and oxidative stress markers

For antioxidant enzymatic assays, skeletal red and white muscle and liver samples were homogenized in phosphate buffer (pH 7.4). Superoxide dismutase (SOD) activity was assessed using a commercial assay kit (19,160-1KT-F, Sigma) according to the manufacturer’s instructions. Catalase (CAT) activity was measured using hydrogen peroxide (30%) as substrate (Claiborne 1985). Glutathione-S-transferase (GST) activity was determined by using 1-chloro-2,4-dinitrobenzene acting as substrate (Habig et al. 1974). The activities of glutathione reductase (GR) and glutathione peroxidase (GPx) were determined based on the oxidation of NADPH (Mohandas et al. 1984; Cribb et al. 1989). The enzymatic activities were expressed in relation to the analyzed protein concentration in each sample using bovine serum albumin as a standard (Bradford 1976). Lipid peroxidation (LPO) level was quantified using thiobarbituric acid as substrate and expressed as nmol thiobarbituric acid reactive substances (TBARS) per g of tissue (Ohkawa et al. 1979). Total glutathione and oxidized glutathione (GSSG) levels were measured by the reaction of reduced glutathione (GSH) with 5,5′-dithiobis-(2-nitrobenzoic acid; DTNB) according to the protocol of Baker et al. (1990). To calculate the GSSG content, 2-vinyl-pyridine was used to eliminate free thiols present in the samples (Griffith 1980). The GSH content was then calculated by subtracting the amount of GSSG from the total glutathione levels determined. The GSH and GSSG levels were expressed as nmol of conjugated TNB formed per min per mg of protein. Absorbance changes in all assays were measured at 25 °C in a microplate reader (BioTek Instruments, Inc.).

### Statistical analysis

Results were expressed as mean ± standard error of the mean (SEM). Data were analyzed using one-way ANOVA followed by Tukey’s test to assess differences between the experimental conditions. The normality of the data was determined using the Shapiro–Wilk test and the homogeneity of variance using the Levene test. Non-normally distributed data were log-transformed to perform parametric tests, and if data did not meet parametric assumptions, a non-parametric Kruskal–Wallis test followed by a Dunn’s multiple comparison test was used. The significance level used was *P* < 0.05. All statistical analyses were performed with GraphPad Prism 8.0 (GraphPad Software Inc.).

## Results

### Blood cell count and plasma stress markers

**Fig. 1:**
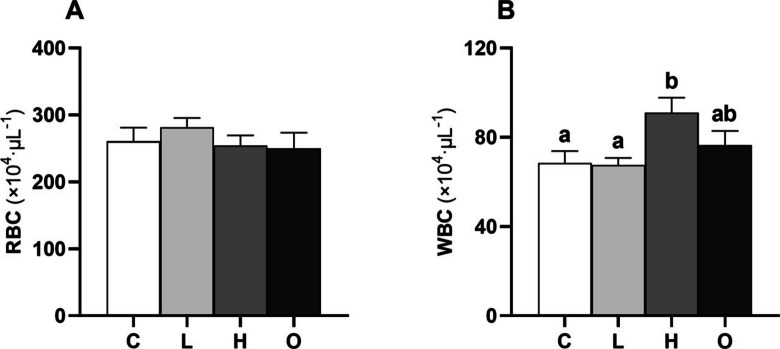
Total red (**A** RBC) and white blood cell (**B** WBC) counts of European seabass (*Dicentrarchus labrax*) subjected to different conditions: control (C); steady low (L); steady high (H); and oscillating (O) swimming speeds. Bars represent mean ± SEM (*n* = 8). Different letters indicate significant variations between the experimental groups (one-way ANOVA, *P* < 0.05)

**Fig. 2:**
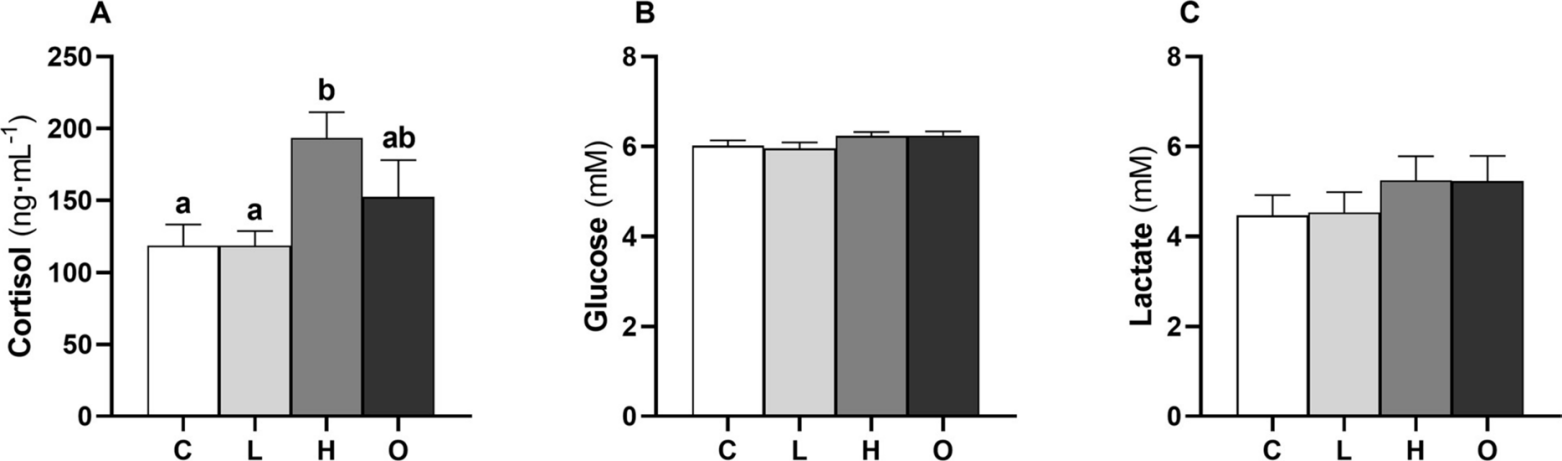
Cortisol (**A**), glucose (**B**), and lactate (**C**) concentrations in the plasma of European seabass (*Dicentrarchus labrax*) subjected to different conditions: control (C); steady low (L); steady high (H) and oscillating (O) swimming speeds. Bars represent mean ± SEM (*n* = 8). Different letters indicate significant differences between the experimental groups (one-way ANOVA, *P* < 0.05)

No variations were observed in total RBC counts between the experimental groups (Fig. 1A), whilst the number of WBC increased in the H group compared to the C and L conditions (*P* = 0.023) (Fig. 1B). In addition, cortisol concentration was higher in the H group than in the C and L groups (*P* = 0.013) (Fig. 2A), while plasma glucose (Fig. 2B) and lactate (Fig. 2C) concentrations remained unchanged.

### Innate immune parameters in plasma and skin mucus

Natural hemolytic complement, lysozyme, and peroxidase activities did not show variations in plasma (Table 2) and skin mucus (Table 3). The bactericidal activity against *V. anguillarum* and *P. damselae* in skin mucus did not change among experimental groups (Table 3).

**Table 2:** Natural hemolytic complement, lysozyme, and peroxidase activities in plasma of European seabass (*Dicentrarchus labrax*) subjected to different conditions: control (C); steady low (L); steady high (H), and oscillating (O) swimming speeds. The values represent mean ± SEM (*n* = 8). No significant differences were detected between groups (natural hemolytic complement: one-way ANOVA, *P* > 0.05; lysozyme and peroxidase: Kruskal–Wallis test, *P* > 0.05)

	Experimental groups			
	C	L	H	O
Natural hemolytic complement (ACH_50_**⋅**mL^−1^)	62.13 ± 9.46	66.08 ± 5.49	60.64 ± 8.63	61.52 ± 10.07
Lysozyme (μg**⋅**mL^−1^ HEWL)	26.36 ± 1.87	29.59 ± 2.20	26.10 ± 1.85	27.61 ± 1.47
Peroxidase (U**⋅**mL^−1^)	41.69 ± 1.45	42.80 ± 1.65	46.48 ± 2.29	46.38 ± 2.54

**Table 3:** Lysozyme, peroxidase, and bactericidal activities in the skin mucus of European seabass (*Dicentrarchus labrax*) subjected to different conditions: control (C); steady low (L); steady high (H), and oscillating (O) swimming speeds. The values represent mean ± SEM (*n* = 8). No significant differences were detected between groups (lysozyme and bactericidal activities: one-way ANOVA, *P* > 0.05; peroxidase: Kruskal–Wallis test, *P* > 0.05)

	Experimental groups			
	C	L	H	O
Lysozyme (μg**⋅**mL^−1^ HEWL)	6.67 ± 0.23	6.48 ± 0.61	7.13 ± 0.31	6.68 ± 0.085
Peroxidase (U**⋅**mL^−1^)	15.52 ± 0.32	16.73 ± 0.43	16.28 ± 0.32	16.81 ± 0.28
Bactericidal activity (%)				
*Vibrio anguillarum*	41.99 ± 5.18	48.72 ± 7.31	35.26 ± 6.74	38.91 ± 3.25
*Photobacterium damselae*	79.08 ± 2.06	77.24 ± 2.22	72.99 ± 1.68	73.32 ± 1.60

### Gene expression

**Fig. 3:**
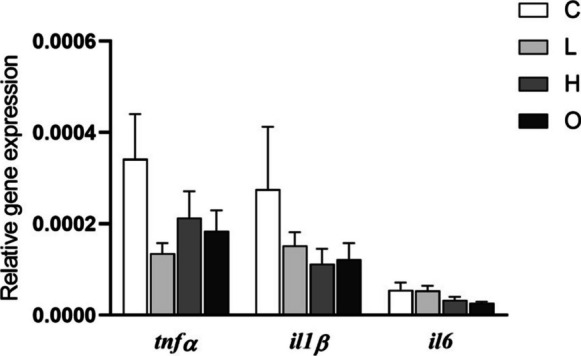
Relative expression of cytokine (tumor necrosis factor-alpha, *tnfα*; interleukin 1 beta, *il1β*; and interleukin 6, *il6*) genes in the head-kidney of European seabass (*Dicentrarchus labrax*) subjected to different swimming conditions: control (C); steady low (L); steady high (H); and oscillating (O) swimming speeds. Gene expression was analyzed by real-time qPCR and normalized to housekeeping gene elongation factor 1-alpha. The bars represent mean ± SEM (*n* = 8). No significant differences were detected between groups (*tnfα* and *il6*: one-way ANOVA, *P* > 0.05; *il1β*: Kruskal–Wallis test, *P* > 0.05)

**Fig. 4:**
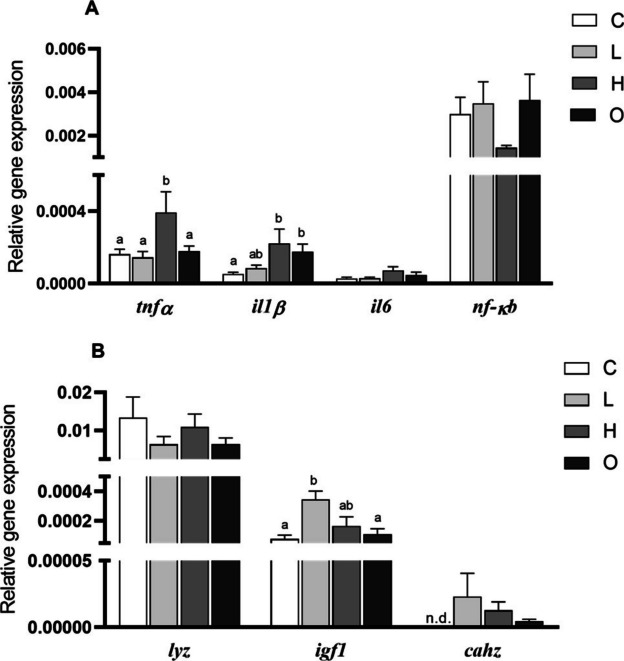
Relative expression of cytokines and inflammatory mediators (**A** tumor necrosis factor-alpha, *tnfα*; interleukin 1 beta, *il1β*; interleukin 6, *il6*; nuclear factor kappa B, *nfκb*) and immune and developmental genes (**B** lysozyme, *lyz*; insulin growth factor 1, *igf1*; carbonic anhydrase, *cahz*) in gills of European seabass (*Dicentrarchus labrax*) subjected to different swimming conditions: Control (C); steady low (L); steady high (H); and oscillating (O) swimming speeds. Gene expression was analyzed by real-time qPCR and normalized to housekeeping gene elongation factor 1-alpha. Bars represent mean ± SEM (*n* = 8); n.d. = not detected. Different letters indicate significant differences between experimental groups (one-way ANOVA, *P* < 0.05)

No variations were found in the expression of genes analyzed (*tnfα*, *il1β*, and *il6*) in the HK (Fig. 3). Similarly, no variations were observed in the expression of *il6* and *nfκb* genes in the gills of fish from all experimental groups. Nevertheless, the expression of the *tnfα* gene was up-regulated in the gills of fish from the H group compared to the other experimental groups (*P* = 0.016). The expression of *il1β* gene was up-regulated in the gills of fish from the H and O groups compared to the values found in the fish from C group (*P* = 0.003) (Fig. 4A). However, the expression of *lyz* and *cahz* genes did not show variations in the gills, whilst the expression of *igf1* gene was up-regulated in the L group compared to the C and O groups (*P* = 0.010) (Fig. 4B).

**Fig. 5:**
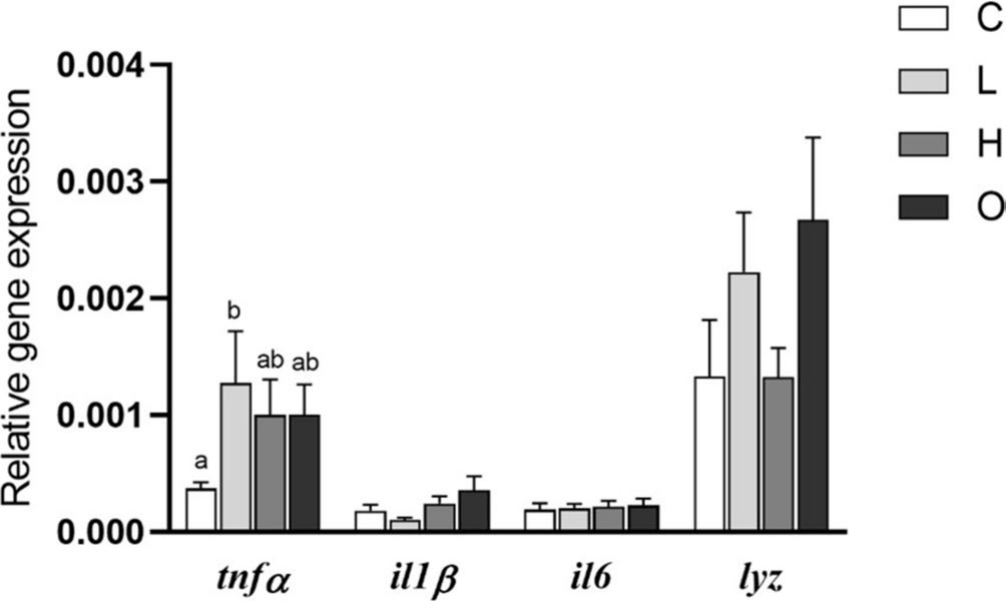
Relative expression of cytokines (tumor necrosis factor-alpha, *tnfα*; interleukin 1 beta, *il1β;* and interleukin 6, *il6*) and immune-related (lysozyme; *lyz)* genes in the skin of European seabass (*Dicentrarchus labrax*) subjected to different swimming conditions: control (C); steady low (L); steady high (H); and oscillating (O) swimming speeds. Gene expression was analyzed by real-time qPCR and normalized to housekeeping gene elongation factor 1-alpha. Bars represent mean ± SEM (*n* = 8). Different letters indicate significant differences between the experimental groups (one-way ANOVA, *P* < 0.05)

In skin, the expression of the *tnfα* gene was up-regulated in the fish from the L group compared to the C group (*P* = 0.042), whilst no variations were observed among groups in the expression of *il1β*, *il6*, and *lyz* genes (Fig. 5).

### Metabolic and antioxidant enzymes and oxidative stress markers

**Fig. 6:**
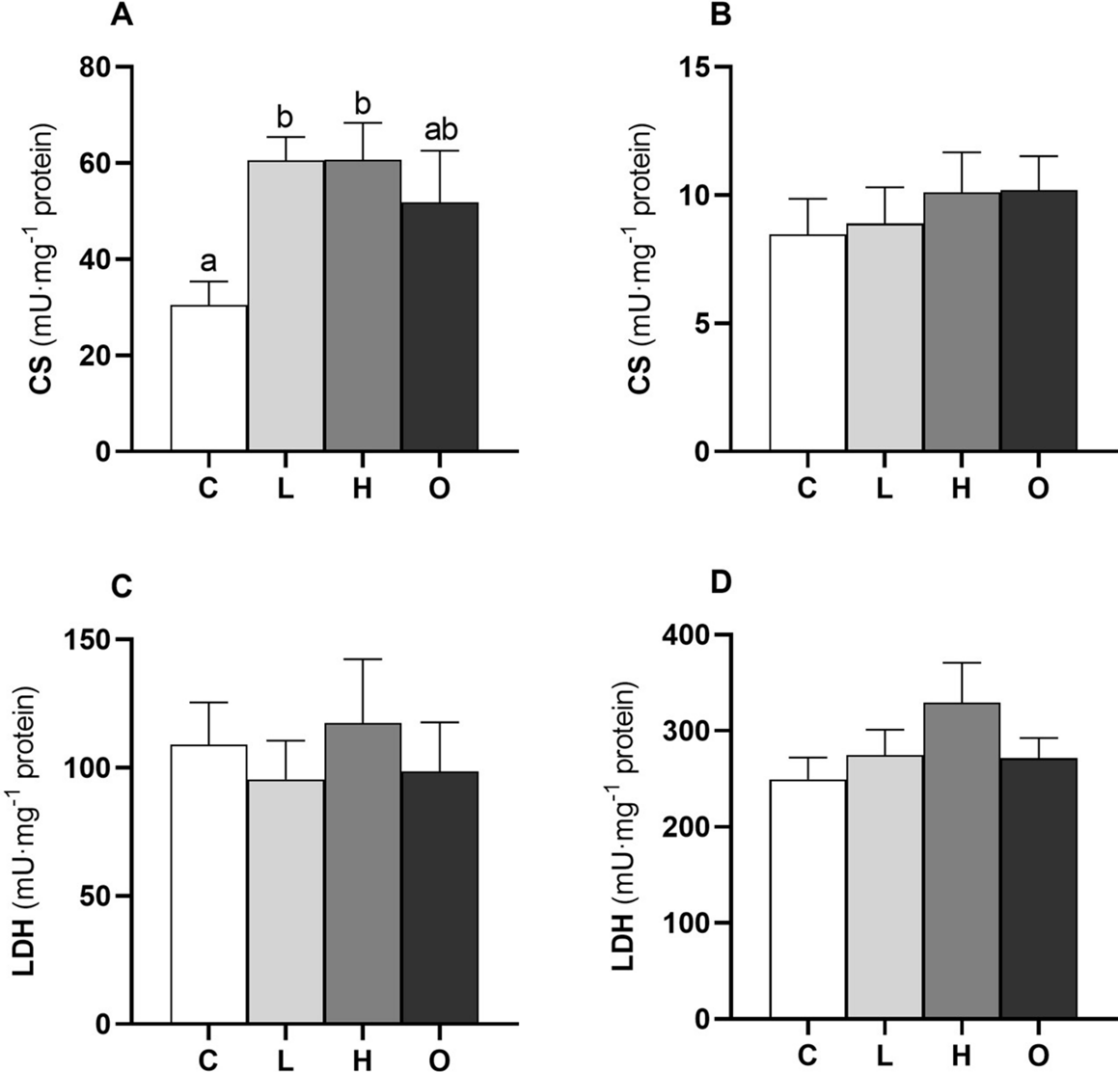
Metabolic enzyme activities in the red (**A** and **C**) and white muscle (**B** and **D**) of European seabass (*Dicentrarchus labrax*) subjected to different swimming conditions: control (C); steady low (L); steady high (H); and oscillating (O) swimming speeds. CS, citrate synthase, and LDH, lactate dehydrogenase activities. Bars represent mean ± SEM (*n* = 8). Different letters indicate significant differences between the experimental groups (one-way ANOVA, *P* < 0.05)

The activity of CS in the red muscle was higher in groups L and H than in the C group (*P* = 0.011) (Fig. 6A), whilst no variations were found for CS activity in the white muscle between the experimental groups (Fig. 6B). There were also no differences in LDH activity between the four experimental groups, neither in the red muscle (Fig. 6C) nor in the white muscle (Fig. 6D).

On the other hand, the activities of SOD (*P* = 0.024) and CAT (*P* = 0.029) were higher in the red muscle of the H group compared to the control group. In addition, LPO levels were increased in the H group compared to the C group (*P* = 0.025). The GSH content (*P* = 0.039) and the GSH: GSSG ratio (*P* = 0.020) were also increased in the L group compared to the other groups (Table 4).

**Table 4:** Activities of antioxidant enzymes (superoxide dismutase, SOD; catalase, CAT; glutathione-S-transferase, GST; glutathione reductase, GR; glutathione peroxidase, GPx) and oxidative stress markers (lipid peroxidation, LPO; reduced glutathione, GSH; oxidized glutathione, GSSG) in the red skeletal muscle of European seabass (*Dicentrarchus labrax*) subjected to different swimming conditions: control (C); steady low (L); steady high (H); and oscillating (O) swimming speeds. Different letters indicate significant differences between the experimental groups (one-way ANOVA, *P* < 0.05)

	Experimental groups			
	C	L	H	O
SOD (U**⋅**mg^−1^ protein)	7.68 ± 0.56^a^	8.19 ± 0.88^a^	13.05 ± 1.34^b^	10.39 ± 0.79^ab^
CAT (U**⋅**mg^−1^ protein)	27.64 ± 3.10^a^	26.09 ± 2.69^a^	34.94 ± 2.57^b^	30.11 ± 4.33^ab^
GST (mU**⋅**mg^−1^ protein)	3.18 ± 0.09	3.09 ± 0.08	3.11 ± 0.10	3.04 ± 0.18
GR (mU**⋅**mg^−1^ protein)	3.55 ± 1.10	3.16 ± 0.95	3.37 ± 1.14	3.41 ± 1.36
GPx (U**⋅**mg^−1^ protein)	3.69 ± 0.16	3.39 ± 0.22	3.51 ± 0.24	3.59 ± 0.29
LPO (nmol**⋅**TBARS g^−1^ tissue)	64.59 ± 3.10^a^	79.31 ± 3.34^ab^	85.16 ± 7.34^b^	68.08 ± 5.18^ab^
GSH (nmol**⋅**TNB min^−1^ mg^−1^ protein)	1.28 ± 0.09^a^	1.83 ± 0.10^b^	1.20 ± 0.21^a^	1.33 ± 0.18^b^
GSSG (nmol**⋅**TNB min^−1^ mg^−1^ protein)	0.85 ± 0.08	0.79 ± 0.05	0.76 ± 0.04	0.85 ± 0.07
GSH: GSSG ratio	2.84 ± 0.61^a^	4.15 ± 0.88^b^	3.32 ± 0.61^a^	3.13 ± 0.32^a^

Furthermore, no differences in the activities of antioxidant enzymes in white muscle were detected (Table 5). Nevertheless, the GSSG content (*P* = 0.005) and GSH: GSSG ratio (*P* = 0.015) increased in all swimming groups compared to the C group. The activity of antioxidant enzymes and oxidative stress markers in the liver did not show differences between groups (Table S1, Supplementary information).

**Table 5:** Activities of antioxidant enzymes (superoxide dismutase, SOD; catalase, CAT; glutathione-S-transferase, GST; glutathione reductase, GR; glutathione peroxidase, GPx) and oxidative stress markers (lipid peroxidation, LPO; reduced glutathione, GSH; oxidized glutathione, GSSG) in the white skeletal muscle of European seabass (*Dicentrarchus labrax*) subjected to different swimming conditions: control (C); steady low (L); steady high (H); and oscillating (O) swimming speeds. Different letters indicate significant differences between the experimental groups (one-way ANOVA, *P* < 0.05)

	Experimental groups			
	C	L	H	O
SOD (U**⋅**mg^−1^ protein)	4.16 ± 0.10	4.22 ± 0.07	4.09 ± 0.09	4.16 ± 0.11
CAT (U**⋅**mg^−1^ protein)	8.67 ± 0.41	8.16 ± 0.33	8.46 ± 0.18	8.39 ± 0.28
GST (mU**⋅**mg^−1^ protein)	2.30 ± 0.08	2.44 ± 0.10	2.36 ± 0.13	2.29 ± 0.22
GR (mU**⋅**mg^−1^ protein)	1.86 ± 0.15	1.80 ± 0.12	2.12 ± 0.17	1.87 ± 0.11
GPx (U**⋅**mg^−1^ protein)	2.51 ± 0.15	2.44 ± 0.30	2.48 ± 0.27	2.55 ± 0.29
LPO (nmol**⋅**TBARS g^−1^ tissue)	39.03 ± 3.44	38.49 ± 2.80	39.84 ± 2.46	38.68 ± 2.41
GSH (nmol TNB min^−1^ mg^−1^ protein)	0.87 ± 0.14	0.74 ± 0.10	0.81 ± 0.14	0.99 ± 0.11
GSSG (nmol TNB min^−1^ mg^−1^ protein)	1.10 ± 0.07^a^	0.78 ± 0.06^b^	0.79 ± 0.04^b^	0.82 ± 0.07^b^
GSH: GSSG ratio	1.25 ± 0.15^a^	2.21 ± 0.29^b^	2.26 ± 0.23^b^	2.31 ± 0.32^b^

## Discussion

### High swimming speed is associated with increased plasma cortisol and WBC counts

The beneficial effects of induced swimming on lowering stress levels in fish have been postulated, particularly in salmonids, suggested by reduced circulating cortisol levels upon swimming activity (Woodward and Smith 1985; Boesgaard et al. 1993; Herbert et al. 2011). However, this response appears to be highly dependent on the fish species and the intensity and duration of the induced swimming activity. For example, some studies have shown that induced swimming leads to increased plasma cortisol concentrations in Arctic charr (*Salvelinus alpinus*) (Christiansen et al. 1991) and rainbow trout (*Oncorhynchus mykiss*) (Milligan 1997).

This study assessed physiological parameters in European seabass subjected to different swimming conditions, by including a control group that was not induced to swim. This control group experienced the same manipulations as the other fish in different treatments, except for the induced swimming. All experimental fish were given 24 h to acclimate after being transferred to the swimming flumes, which were set to a very low water flow to ensure proper water renewal and oxygenation under reduced lighting conditions. This setup was aimed at facilitating recovery from manipulations. It may be argued that the experimental design applied in seabass did not consider comparisons with plasma cortisol levels at a basal state to have a more complete appraisal of stress response in this species. Despite this potential limitation, plasma cortisol levels measured in the control group in this study ranged between 72 and 170 ng⋅mL⁻^1^, which is consistent with values reported in European seabass under basal conditions. Recent meta-analyses reported basal plasma cortisol levels in European seabass to range from 66 to 110 ng⋅mL⁻^1^, while post-stress levels typically range from 311 to 461 ng⋅mL⁻^1^ in this species (Alfonso et al. 2023; Samaras 2023). Therefore, the experimental conditions applied in this study resulted in effective recovery from any stress that may be associated with transferring and/or holding fish to the swimming flumes.

In the current study, plasma cortisol levels were significantly higher in fish subjected to steady swimming at 2.2 BL⋅s^−1^ (H group), when compared to control and fish subjected to steady swimming at 0.8 BL⋅s^−1^ (L group), which may indicate that 6 h of high-intensity swimming may have caused an activation of the hypothalamus-pituitary-interrenal (HPI) axis, leading to the release of cortisol (Faught and Schaaf 2024), and most likely the release of catecholamines from chromaffin cells into the circulation (Reid et al. 1998). A previous study with gilthead seabream (*Sparus aurata*) found that 6 h of induced swimming at 2.3 BL⋅s^−1^ did not affect the plasma cortisol levels in that species (Espírito-Santo et al. 2023). In comparison, the results obtained in this study highlight the lower stress resistance previously reported in European seabass (Samaras et al. 2018), suggesting that swimming at 2.2 BL⋅s^−1^ may not be a recommended swimming condition to be applied in European seabass in aquaculture settings, as it may result in decreased welfare. Interestingly, it has been described that potential stressors can influence the immune function (Guo and Dixon 2021). In fact, in our study, a higher number of WBC was observed in the H group, which can also occur during acute stress responses (Tort 2011). However, no variations in the innate immune parameters analyzed were observed. It is possible to consider that a longer swimming period at high-intensity swimming may be needed to reveal an impact on immune status.

### High swimming speed up-regulated cytokines in gills and skin

Interestingly, gene expression analysis revealed an up-regulation of cytokine genes (*tnfα* and *il1β*) in gills in the H condition. The role of cytokines in fish has been studied in recent years, being involved in several parameters of the immune system, regulating inflammation and cell proliferation (Zou and Secombes 2016). Furthermore, the modulation of cytokines expression upon physical activity has been described in mammals, being involved, for example, in muscle tissue repair and inflammation upon high-intensity exercise loads (Cerqueira et al. 2020; Małkowska and Sawczuk 2023). In fish, studies with Atlantic salmon demonstrated the modulation of inflammatory cytokines upon different induced swimming conditions (Castro et al. 2011, 2013). TNF-α (tumor necrosis factor α) and IL-1β (interleukin 1 β) are cytokines that, upon release, start a cascade of events leading to inflammation (Secombes et al. 2001). Upon secretion of such cytokines, in combination with the retraction of endothelial cells to increase blood through vasodilation, the recruitment of leukocytes to the site of inflammation is facilitated (Roca et al. 2008). Also, cytokines have been described to be produced and released at the first stage of the stress response (Teles, et al. 2011; Duque and Descoteaux 2014). For instance, it has been demonstrated that the expression of inflammatory cytokines in gills is also altered by induced salinity stress in species such as spotted scat (*Scatophagus argus*) (Zhong et al. 2021), clown knifefish (*Notopterus chitala*) (Moniruzzaman et al. 2022), and silvery pomfret (*Pampus argenteus*) (Li et al. 2020). Besides the maintenance of gas and ionic changes, the gills are also an immune-competent tissue, comprising a mucosal-associated lymphoid tissue (Koppang et al. 2015; Xu et al. 2016). Also, in a recent study with gilthead seabream, 6 h of oscillating swimming speeds (between 0.2 and 0.8 BL⋅s^−1^) resulted in the up-regulation of *tnfα*, *il1β*, and *il6* genes in gills, suggesting an inflammatory response in that tissue at relative low but oscillating swimming speeds, substantially beneath the described optimal swimming speed (*U*_*opt*_) for that species and revealing a modulation in gills cytokines induced by swimming (Espírito-Santo et al. 2023). The up-regulation of *tnfα* and *il1β* genes in the gills of European seabass swimming at a relatively high speed compared to the other experimental groups reveals a possible inflammation response occurring in this tissue. These results are in accordance with the increased number of WBC and plasma cortisol levels observed in fish from the same group, indicating possible recruitment of leukocytes from circulation to the site of inflammation, in response to stress. The stress-induced inflammation response in gills upon induction of 2.2 BL⋅s^−1^ emphasizes the vulnerability of this tissue, potentially explained by the increased gas exchange demand in European seabass juveniles swimming in such a condition. Nevertheless, long-term induction of swimming activity has been shown to trigger remodeling of the gills by increasing their surface area in goldfish (*Carassius auratus*) (Perry et al. 2012) and crucian carp (*Carassius carassius*) (Brauner et al. 2011). Interestingly, scombrids are described to have higher gill surface areas and thinner gill epithelium compared to most teleosts, increasing the efficiency of gas exchange, related to their intrinsic high swimming performance (Bushnell and Jones 1994).

However, data on how different induced swimming speeds may affect the form and function of gills are scarce. It may be possible that an increasing oxygen demand triggered by high swimming speeds may be related to observed changes at gene transcription levels for an inflammatory response in gills, which may result in vasodilation in this tissue to facilitate gas exchange. Interestingly, the L condition triggered an up-regulation of the *igf1* gene in gills. The role of IGF-1 (insulin growth factor 1) in gills has been described to be involved in the smoltification process in salmonids, modulating gill morphology for adaptation to seawater (Breves et al. 2017; Cui et al. 2022). Still, it is interesting that the up-regulation of the *igf1* gene did not occur at the higher swimming speed, suggesting that such adaptation may not be directly correlated with the swimming intensity but rather speed-specific.

In skin, the expression of *tnfα* was up-regulated in the L group compared to the C group. The role of skin in swimming is yet to be elucidated, although some studies have suggested its importance in swimming efficiency, mainly through its hydrodynamic properties (Vernerey and Barthelat 2014). In some species, TNFα is described to be involved in the skin immune response. For example, Campos-Sánchez et al. (2021) observed an up-regulation of the *tnfα* gene in the skin of gilthead seabream after 6 h of an injection of carrageenin, suggesting the role of TNF-α in the activation of endothelial cells and recruitment of granulocytes to the site of injection. Interestingly, in a subsequent study, Campos-Sánchez et al. (2022) described an increased number of mucus-secreting cells in the skin of gilthead seabream following an induced inflammation by carrageenin. It is possible that the up-regulation of the *tnfα* gene in the skin of fish swimming under the H condition could be associated with changes in skin mucus production, possibly affecting swimming performance. Curiously, yellow European eels (*Anguilla anguilla*) swimming for 0.3 BL⋅s^−1^ for 7 h also revealed an inflammatory process in the skin with an up-regulation of the *il1β* gene compared to fish that have not been induced to swim (Espírito-Santo 2024b). It has been described that remodeling of skin mucus properties such as viscoelasticity improves swimming efficiency through drag reduction (Sagnes et al. 2000). So, further studies are needed to elucidate the relationship that induced swimming may have on skin and skin mucus properties, taking into consideration functional aspects.

Concerning the HK, no variations in the gene expression of cytokines were observed. The HK is the primary lymphatic immune organ in fish, playing a crucial role in the coordination of innate immune function, susceptible to external environmental changes (Mokhtar et al. 2023). Nevertheless, while some studies shed light on the relationship between certain swimming speeds and the modulation of innate immune parameters, the role of the HK in regulating the immune status upon swimming activity is yet to be elucidated.

### Swim-related changes in metabolic enzyme activities and antioxidant status

It has been shown that the growth rate of some species is improved at their *U*_*opt*_, i.e. the lowest energy expenditure during swimming (Palstra and Planas 2011). Furthermore, swimming training is associated with mitochondrial remodeling in the skeletal muscle of fish (Morash et al. 2014; Blasco et al. 2015; Pengam et al. 2021). Similarly, swimming can rapidly alter metabolic pathways due to the increased energy demands of skeletal muscle during high-intensity swimming, resulting in increased oxygen delivery to the contracting muscle (Weber and Haman 1996; Magnoni et al. 2014; Gerry and Ellerby 2014).

In this study, CS activity was higher in red muscle in all induced swimming conditions, whereas no significant differences were found in white muscle. Citrate synthase is a key enzyme that catalyzes the first of a series of Krebs cycle reactions and is an indicator of aerobic metabolism (Childress and Somero 1979). Therefore, the higher CS activity in red muscle is indicative of increased aerobic metabolism and is expected during swimming activity, also due to higher mitochondrial content and vascularization (Dalziel et al. 2005). On the other hand, LDH activity in red and white muscle also did not change between the groups. Lactate dehydrogenase is a glycolytic enzyme involved in anaerobic metabolism (Childress and Somero 1979).

Given the gap in knowledge on the effect that swimming activity may have on the redox status of fish, we analyzed the effects of different swimming conditions on oxidative stress markers in red and white skeletal muscle and liver. The existing studies on this topic have shown different results depending on the species and intensity of exercise, which limits the broad applicability to other species. For example, a study by Mortelette et al. (2010) showed that induced swimming at speeds of 1.3 to 1.8 BL⋅s^−1^ leads to increased production of reactive oxygen species (ROS) in the skeletal muscle of the silver European eels, potentially causing structural damage. Consequently, the increased ROS production together with a redox imbalance may lead to lipid and protein oxidation, which in turn results in oxidative stress. Furthermore, Mortelette et al. (2010) reported that silver European eels induced to swim showed lower CAT, SOD, and GPx activities and lower LPO levels in red muscle, compared to non-swimming fish. Furthermore, Sánchez-Moya et al. (2022) demonstrated that gilthead seabream fingerlings swimming at 2.5 BL⋅s^−1^ presented lower LPO levels in white muscle, compared to fish that were not induced to swim.

Nonetheless, the modulation of antioxidant markers in red and white skeletal muscle was observed in this study under different swimming conditions. The activities of SOD and CAT were increased in the red muscle of fish from the H group. Superoxide dismutase is responsible for the conversion of superoxide, a ROS, into hydrogen peroxide, which can subsequently be converted into oxygen and water by CAT (Vélez-Alavez et al. 2015). In addition, LPO levels increased in the red muscle of the same group, indicating oxidative stress, which may suggest that the increase in both SOD and CAT activities was not sufficient to counteract the ROS that could be generated by swimming. A similar observation was described by Espírito-Santo et al. (2024a) in gilthead seabream, where oscillating swimming regime (varying between 0.8 and 2.3 BL⋅s^−1^) resulted in higher GPx and CAT activities together with increased levels of LPO compared to non-swimming and steady (0.8 and 2.3 BL⋅s^−1^) swimming conditions. European seabass uses a subcarangiform swimming mode and recruits the red muscle for sustained swimming, through aerobic metabolism (Bone 1978). Sustained swimming promotes a shift towards a more aerobic muscle profile, enhancing mitochondrial growth and activity of antioxidant enzymes (McClelland et al. 2005; Magnoni et al. 2014; Pengam et al. 2021; Perelló-Amorós et al. 2021, 2024). The results in this study highlight how the red muscle may be prone to oxidative stress when European seabass juveniles are induced to swim at 2.2 BL⋅s^−1^, which can potentially impair the function of the red muscle and impose further challenges for European seabass to cope with different swimming speeds in long-term trials.

Claireaux et al. (2006) defined a positive correlation between swimming speed and oxygen consumption in European seabass. Considering the results of this study, it is plausible to infer that swimming at 2.2 BL⋅s^−1^ (H group) for 6 h may lead to an increase in ROS production, which may affect the redox status in red muscle. Interestingly, induced swimming at 0.8 BL⋅s^−1^ (L group) led to an increase in the GSH: GSSG ratio, which is mainly due to a higher content of GSH in the red muscle. A higher GSH: GSSG ratio is considered a biomarker for an effective redox status as GSH is responsible for regulating the scavenging of ROS and their resultant products (Srikanth et al. 2013). Upon reaction with oxidizing agents, GSH is converted to GSSG, and this form of glutathione is later reduced by the enzyme GR, thereby regenerating GSH (Srikanth et al. 2013). These results suggest an improved redox status of the red muscle of European seabass when induced to swim at the specific conditions applied in the low-speed (L) group.

Interestingly, in all swimming conditions, fish presented lower levels of GSSG in white muscle, compared to non-swimming fish, resulting in higher GSH: GSSG ratio. These results are consistent with a study conducted in gilthead seabream under similar conditions (Espírito-Santo et al. 2024a), which has a similar swimming mode as European seabass. The white muscle, which comprises most of the muscle mass, relies on anaerobic metabolism and is utilized in short, high-intensity sprints (Bone 1978; Jayne and Lauder 1993). However, it is not excluded that white muscle can also partially be recruited in fish swimming at speeds just below the critical swimming speed (Lindsay 1978). Given the results obtained, it is plausible to infer that the antioxidant status of white muscle is increased in European seabass juveniles when induced to swim in all conditions applied compared to the control group. These results are of great importance, not only for understanding the role of the white muscle during swimming activity in European seabass, regardless of its involvement in the locomotion of the fish, but also to better understanding the hyperplasia process described in other studies, upon induced swimming at specific speeds (Huang et al. 2021).

The liver, on the other hand, showed no changes in the oxidative stress parameters analyzed. Fish liver is a highly metabolically active tissue, key in mitigating generated ROS, and highly involved in bio-transformation (Birnie‐Gauvin et al. 2017; Bruslé and Anadon 2017). Due to its role in regulating energy balance and detoxification, the proper regulation of its redox status is therefore crucial for fish homeostasis. In gilthead seabream, higher hepatic GSH levels were observed in fingerlings swimming at 2.5 BL⋅s^−1^ for 6 weeks (Sánchez-Moya et al. 2022) and in juveniles swimming for 6 h at 0.8 and 2.2 BL⋅s^−1^ (Espírito-Santo et al. 2024a) compared to non-swimming fish. However, the modulation of hepatic redox status by swimming activity should depend on the energy allocation and regulation occurring in each species, emphasizing the variability in the effects of swimming activity on the redox status of different fish species.

## Conclusions

Several studies have investigated the swimming performance of European seabass. However, this is the first study to date to investigate the effects of induced swimming under different conditions on the immune and antioxidant status in European seabass. The innate immune parameters in plasma and skin mucus of juvenile seabass were not altered by any of the different conditions applied. However, after 6 h of steady swimming at 2.2 BL⋅s^−1^, seabass displayed higher WBC counts and circulating cortisol levels. These changes may be correlated to the inflammatory response in gills, shown at the gene expression level, as an effect of increased oxygen demand triggered by swimming. In addition, the results of oxidative stress factors revealed an improved redox status (higher GSH: GSSG ratio) in red muscle in fish swimming at 0.8 BL⋅s^−1^ and in white muscle in all swimming conditions. Nevertheless, steady swimming at 2.2 BL⋅s^−1^ in red muscle resulted in higher SOD and CAT activities and increased LPO levels, which may indicate the vulnerability of seabass swimming in such conditions. Understanding the physiological effects of induced swimming is of great importance as it may be an important factor to apply swimming exercise regime for stimulating performances and wellbeing of European seabass during aquaculture practices.

## Supplementary Information

Below is the link to the electronic supplementary material.

Supplementary file1 (DOCX 18.1 KB)

## Data Availability

No datasets were generated or analyzed during the current study.
