# Supplementary material for: Induced swimming in European seabass (Dicentrarchus labrax): effects on the stress response, immune, and antioxidant status
Source: Fish Physiol Biochem. 2025 Mar 3;51(2):58. doi: 10.1007/s10695-025-01474-2 (PMC11876196; doi:10.1007/s10695-025-01474-2)
Supplement: Supplementary file 1 — Supplementary file1 (DOCX 18.1 KB) [file 10695_2025_1474_MOESM1_ESM.docx]

**Induced swimming in European seabass (*Dicentrarchus labrax*): effects on the stress response, immune, and antioxidant status**

Carlos Espírito-Santo ^1,2*^, Francisco A. Guardiola ^3^, Rodrigo O. A. Ozório ^2^, Leonardo J. Magnoni ^4^

^1^ Faculty of Sciences, University of Porto, Rua do Campo Alegre, 4069-007 Porto, Portugal.

^2^ Interdisciplinary Centre of Marine and Environmental Research (CIIMAR), University of Porto, Terminal de Cruzeiros do Porto de Leixões, Av. General Norton de Matos s/n, 4450-208, Matosinhos, Portugal.

^3^ Immunobiology for Aquaculture Group, Department of Cell Biology and Histology, Faculty of Biology, Campus Regional de Excelencia Internacional “*Campus Mare Nostrum*”, University of Murcia, 30100, Murcia, Spain.

^4^ The New Zealand Institute for Plant and Food Research Limited, 293 Akersten St. Port Nelson, Nelson, 7010 New Zealand.

*****Corresponding author: Carlos Espírito-Santo, Faculty of Sciences, University of Porto, Rua do Campo Alegre, 4069-007 Porto, Portugal. Email: up202008680@edu.fc.up.pt

**Supplementary information**

Table S1. Antioxidant enzymes and oxidative stress markers in the liver of European seabass subjected to different swimming conditions: control (C); steady low (L); steady high (H) and oscillating (O) speeds. No significant differences were detected between experimental groups (one-way ANOVA, *P* > 0.05).

|  | Experimental groups | | | |
| --- | --- | --- | --- | --- |
|  | C | L | H | O |
| SOD (U⋅﻿mg^-1^ protein) | 8.15 ± 1.71 | 8.73 ± 0.92 | 8.89 ± 1.18 | 8.55 ± 1.36 |
| CAT (U⋅﻿mg^-1^ protein) | 58.06 ± 3.89 | 61.77 ± 6.32 | 60.93 ± 5.30 | 63.55 ± 6.16 |
| GST (mU⋅﻿mg^-1^ protein) | 20.91 ± 2.16 | 19.54 ± 1.91 | 21.49 ± 2.56 | 20.13 ± 3.60 |
| GR (mU⋅﻿mg^-1^ protein) | 7.06 ± 1.84 | 7.91 ± 2.00 | 8.11 ± 1.96 | 7.89 ± 1.36 |
| GPx (U⋅﻿mg^-1^ protein) | 3.94 ± 0.93 | 3.81 ± 0.56 | 3.76 ± 0.60 | 3.79 ± 0.67 |
| LPO (nmol⋅﻿TBARS g^-1^ tissue) | 54.16 ± 8.46 | 49.02 ± 5.98 | 55.66 ± 3.10 | 53.71 ± 8.34 |
| GSH (nmol⋅﻿TNB min^-1^ mg^-1^ protein) | 8.09 ± 2.68 | 8.93 ± 1.51 | 7.57 ± 2.36 | 7.28 ± 2.64 |
| GSSG (nmol⋅﻿TNB min^-1^ mg^-1^ protein) | 2.36 ± 0.12 | 2.45 ± 0.18 | 2.28 ± 0.10 | 2.35 ± 0.21 |
| GSH:GSSG ratio | 3.31 ± 0.84 | 3.60 ± 0.26 | 3.39 ± 0.78 | 3.15 ± 0.61 |
